# Immunometabolism of Macrophages in Bacterial Infections

**DOI:** 10.3389/fcimb.2020.607650

**Published:** 2021-01-29

**Authors:** Gaël Galli, Maya Saleh

**Affiliations:** ^1^University of Bordeaux, CNRS, ImmunoConcEpT, UMR 5164, Bordeaux, France; ^2^Department of Internal Medicine, CHU Bordeaux, Haut Leveque Hospital, Pessac, France; ^3^Department of Medicine, McGill University, Montreal, QC, Canada

**Keywords:** innate immunity, bacteria, inflammation, immunometabolism, infection

## Abstract

Macrophages are important effectors of tissue homeostasis, inflammation and host defense. They are equipped with an arsenal of pattern recognition receptors (PRRs) necessary to sense microbial- or danger-associated molecular patterns (MAMPs/DAMPs) and elicit rapid energetically costly innate immunity responses to protect the organism. The interaction between cellular metabolism and macrophage innate immunity is however not limited to answering the cell’s energy demands. Mounting evidence now indicate that in response to bacterial sensing, macrophages undergo metabolic adaptations that contribute to the induction of innate immunity signaling and/or macrophage polarization. In particular, intermediates of the glycolysis pathway, the Tricarboxylic Acid (TCA) cycle, mitochondrial respiration, amino acid and lipid metabolism directly interact with and modulate macrophage effectors at the epigenetic, transcriptional and post-translational levels. Interestingly, some intracellular bacterial pathogens usurp macrophage metabolic pathways to attenuate anti-bacterial defenses. In this review, we highlight recent evidence describing such host-bacterial immunometabolic interactions.

## Preface

**Figure 1:**
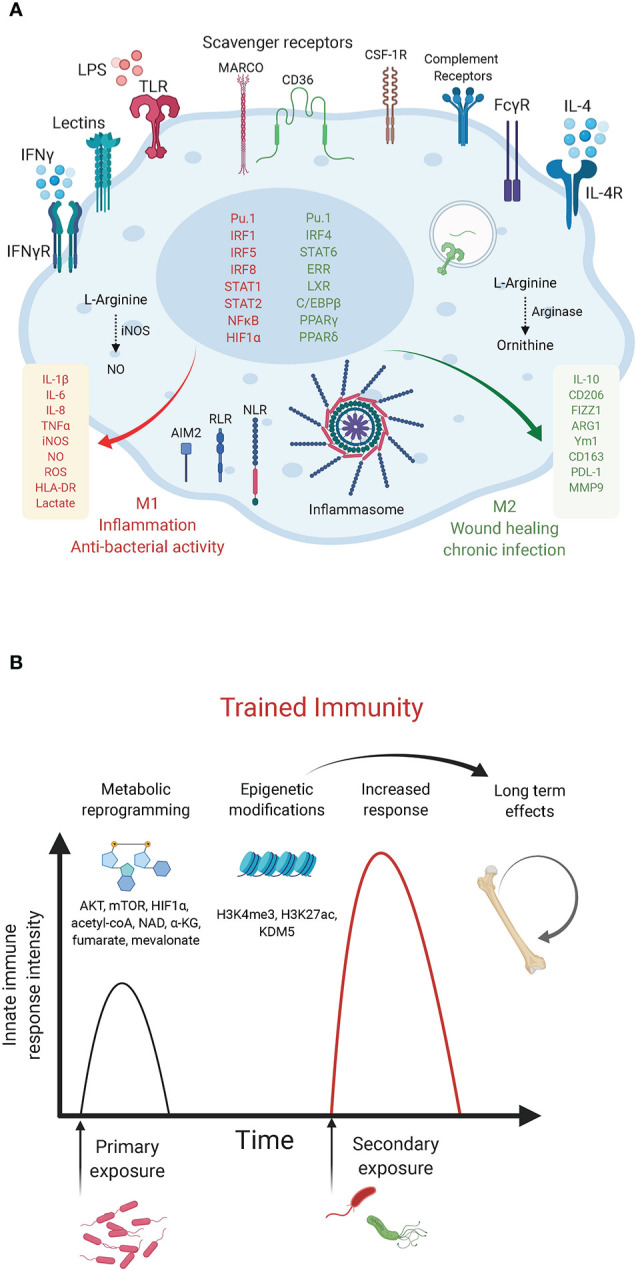
Macrophage innate immunity effectors. **(A)** Macrophage polarization to M1 or M2 is an over-simplified *in vitro* model that does not illustrate the complexity of macrophage ontogeny and phenotypes observed *in vivo*. The main macrophage receptors, transcription factors and markers associated with the M1/inflammatory and bactericidal (red) versus M2/wound healing (green) phenotypes are shown. M1 macrophages shift their metabolism to aerobic glycolysis while M2 macrophages have increased mitochondrial OXPHOS and lipid metabolism. **(B)** Trained immunity in myeloid cells. The graph depicts the enhanced innate immunity response induced upon rechallenge with the same or other microorganisms. Such innate memory is mediated by metabolites (mevalonate, acetyl-coA, NAD, α-KG, fumarate) and metabolic effectors (AKT, mTOR, HIF-1α) that converge on epigenetic (H3K4me3, H3K27ac) reprogramming of myeloid progenitors (Netea et al., 2020).

Derived from hematopoietic precursors, macrophages are central innate immune cells that function in host defense and maintenance of tissue homeostasis. First described by Ilya (Elie) Mechnikov in 1882, macrophages are essentially found in every tissue, seeding the tissue during embryonic development and acquiring specialized organ-specific identities and functions through transcriptional and epigenetic programs governed by factors released by the organ’s stroma (Lavin et al., 2015). In case of perturbations to tissue homeostasis, bone marrow-derived monocytes are recruited from the blood to the affected site where they differentiate into macrophages. Both tissue-resident and monocyte-derived macrophages activate innate and adaptive immunity (Lavin et al., 2015). They act as scavengers that engulf and destroy microbes, particulate matters or altered host cells, while alerting the immune system through the secretion of cytokines, chemokines and lipid mediators. In addition, macrophages contribute to wound healing and tissue repair processes. In contrast, dysregulated activation of macrophages leads to inflammatory tissue damage and inflammatory diseases, cancer promotion, granulomas and chronic infections, atherosclerosis and the metabolic syndrome (Murray and Wynn, 2011). Macrophages express scavenger receptors and immunoglobulin receptors, which promote phagocytosis (Kumar, 2020), antibody-dependent cell phagocytosis (ADCP) and antibody-dependent cell cytotoxicity (ADCC). In addition, they are equipped with germ-line encoded pattern recognition receptors (PRRs) that sense microbial- or danger-associated molecular patterns (MAMPs/DAMPs). Following PRR stimulation, signal transduction cascades converge on the activation of master transcription factors, proteases, and effectors of phagocytosis, allowing a quick innate immune response (Kumar, 2020) (**Figure 1A**). PRR engagement also ensures durable responses through metabolic and epigenetic establishment of an “innate memory”, termed trained immunity. This term coined in 2011 (Netea et al., 2011) refers to the ability of innate immune cells, such as monocytes and macrophages to develop a heightened secondary response, that is rather unspecific, as it occurs following rechallenge by the same or other pathogens. Trained immunity has been demonstrated primarily in studies exploring the response to β-glucan of *Candida albicans* or *Mycobacterium tuberculosis* (*Mtb*) bacillus Calmette-Guérin (BCG). Unlike the classical immunological memory of the acquired immune system, which involves gene recombination events, trained immunity is established by metabolic and epigenetic reprogramming of transcriptional pathways in myeloid progenitors (Netea et al., 2020) (**Figure 1B**).

According to their function in inflammation and host defense versus wound healing and tissue repair, macrophages have been broadly designated as classically activated macrophages or alternatively activated macrophages (also referred to as M1 and M2, respectively). However, such an M1/M2 classification is an *in vitro* paradigm that does not reflect the heterogeneity of macrophages observed *in vivo*, as recently revealed by single cell approaches (Guilliams et al., 2018; Bonnardel and Guilliams, 2018). Previous studies have relied on *in vitro* polarization of murine bone marrow-derived macrophages (BMDM) with bacterial lipopolysaccharide (LPS) + interferon (IFN)γ to obtain the M1 phenotype or interleukin (IL)-4 and/or -13 for M2 polarization. These two macrophage states can be distinguished based on the expression of inducible nitric oxide (NO·) synthase (iNOS) and arginase in M1 and M2 cells, respectively (MacMicking et al., 1997). These markers highlight a primary metabolic difference in the metabolism of arginine into the “killer” molecule NO· in M1 cells or the “repair” metabolite ornithine in M2 cells. Macrophage metabolic adaptations are however not limited to the arginine pathway. As we discuss below, a number of studies have reported a “break” in the TCA cycle and a shift to aerobic glycolysis in M1 macrophages versus a preference for oxidative phosphorylation (OXPHOS) with enhanced glutamine and fatty acid utilization in M2 macrophages. Metabolic intermediates and effectors (e.g. NO·, reactive oxygen species [ROS], TCA derivatives, itaconate, prostaglandins, tryptophan metabolism etc.) were shown to regulate macrophage phenotypes and functions by acting as direct bactericidial agents or through the modulation of innate immunity signaling pathways, leading to the production of cytokines, anti-microbial peptides or tissue repair factors (O’Neill et al., 2016). Here, we focus on recent examples of immunometabolic adaptations following bacterial challenge and of bacterial strategies that target metabolic effectors to modulate host defense mechanisms. The discovery of such immunometabolic interactions provide novel therapeutic entry points to treat immunological disorders and infectious diseases.

## Macrophage Metabolic Rewiring and Associated Functional Outcomes

Prior to delving into macrophage metabolic rewiring, we briefly provide a snapshot of the key cellular bioenergetic pathways described to impact macrophage functions upon bacterial challenge, with a focus on glycolysis and the mitochondrial TCA cycle (Box 1) (O’Neill et al., 2016). Early studies examining macrophage immunometabolism have explored the impact of Toll-like receptors (TLR) engagement, in particular that of TLR4, on metabolic rewiring. Follow-up studies examined live bacterial infections and interrogated the functional outcome of metabolic adaptations on macrophage function. Whether the early metabolic changes impact macrophage polarization or are a consequence of inflammatory signaling is currently debated. Nonetheless, there is ample evidence that metabolic mediators and effectors control both innate immunity and trained immunity in a feedforward manner.

### Nitric Oxide∙ Kick-Starts the Metabolic Rewiring in Lipopolysaccharide-Activated Macrophages

**Figure 2:**
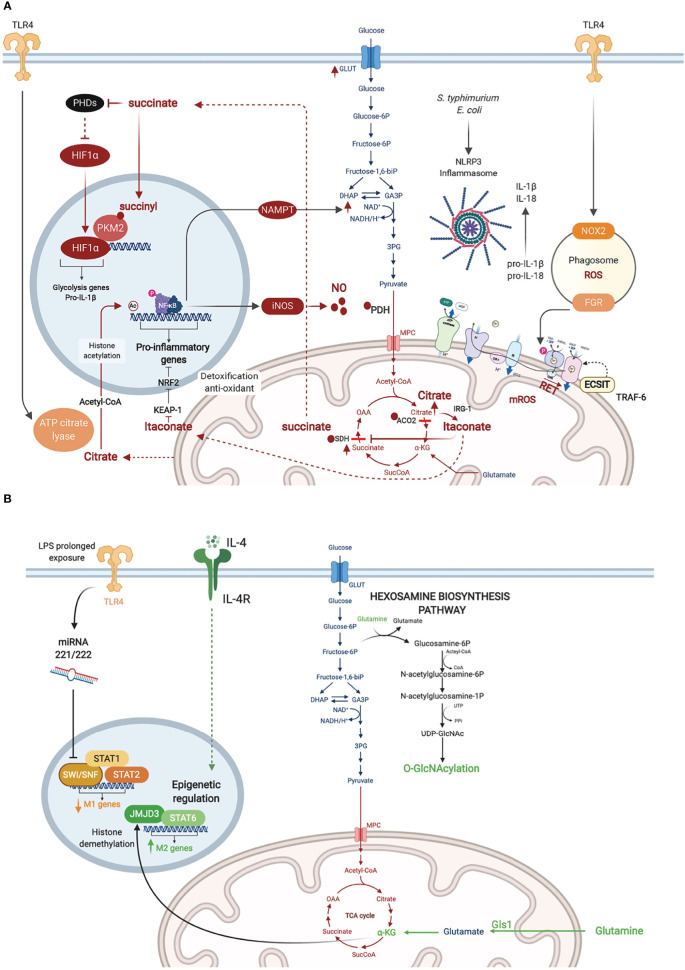
Metabolic adaptations in activated macrophages. **(A)** Inflammatory macrophages are characterized by NO∙ -mediated inhibition of glucose flux in the Tricarboxylic Acid (TCA) cycle. NO∙ inhibits pyruvate dehydrogenase (PDH), aconitase 2 (ACO2), and SDH presumably through cysteine nitrosylation. This results in citrate accumulation and its conversion to itaconate which also blocks SDH. Citrate conversion to acetyl coA by ATP citrate lyase in the cytosol leads to histone acetylation and activation of inflammatory gene loci. SDH inhibition results in succinate accumulation, which inhibits PHDs, stabilizing HIF-1α and enhancing its transcriptional induction of glycolytic and inflammatory genes (e.g. pro-IL-1b). Succinylation of PKM2 leads to its inhibition and translocation to the nucleus where it promotes HIF-1α activity. RET mediated by succinate accumulation leads to ROS production. **(B)** Metabolic adaptations in M2 macrophages are represented in green. Macrophage tolerance in response to prolonged LPS exposure are in yellow.

Macrophages stimulated with LPS upregulate the expression of iNOS and metabolize arginine to produce high levels of NO∙ (MacMicking et al., 1997). Besides its anti-microbial effects, NO∙ has been shown, almost 30 years ago, to inhibit the ETC (Granger and Lehninger, 1982; Stadler et al., 1991; Cleeter et al., 1994). More recently, NO∙ was demonstrated as the main driver of metabolic rewiring in LPS-activated macrophages, as demonstrated using murine BMDM (Palmieri et al., 2020). Previous studies have described two metabolic “breaks” in the TCA cycle in such inflammatory macrophages leading to the accumulation of citrate and succinate. Citrate accumulation was attributed to changes in isocitrate dehydrogenase (IDH1) expression (Jha et al., 2015) and activity (Bailey et al., 2019; De Souza et al., 2019). However, the recent report by Palmieri et al. demonstrated that the break is rather mediated by NO∙-dependent inactivation of aconitase 2 (ACO2) (Palmieri et al., 2020) (**Figure 2A**). NO∙ also inhibited pyruvate dehydrogenase (PDH), potentially through Cysteine nitrosylation of the PDH-E3 subunit (dihydrolipoyl dehydrogenase, DLD), which blunts the entry of pyruvate in the TCA cycle. Cessation of glucose flux thus increases glutamine uptake and its anaplerotic utilization. Concomitantly, NO∙ impairs SDH function (Jha et al., 2015), although this has also been attributed to itaconate-mediated inhibition (Cordes et al., 2016; Lampropoulou et al., 2016). In either case, SDH inhibition leads to succinate accumulation (**Figure 2A**).

### Reactive Oxygen Species and NAD^+^ as Determinants of the Macrophage Inflammatory and Bactericidal Response

In 2011, West et al. reported that stimulation of TLR1, TLR2, and TLR4 on murine BMDM augmented mitochondrial ROS. They demonstrated juxtaposition between phagosome and mitochondria and an interaction between the TLR adaptor TRAF6 and ECSIT (Evolutionarily Conserved Signaling Intermediate In Toll Pathway), a protein involved in CI assembly in the inner mitochondrial membrane. This TLR-induced interaction leads to TRAF6-dependent ubiquitination of ECSIT which alters its localization to the outer mitochondrial membrane resulting in CI disassembly (West et al., 2011) (**Figure 2A**). Depletion of either ECSIT or TRAF6 impaired the ability of murine BMDM to clear intracellular bacteria, such as *Salmonella typhimurium*, supporting a role of CI-derived mROS in antibacterial defense (West et al., 2011). Follow up studies showed that engagement of different TLRs on human monocytes by lysates from different bacteria including *Escherichia coli*, *Mtb* and *Staphylococcus aureus* resulted in a universal increase in aerobic glycolysis, but changes in OXPHOS and lipid metabolism were restricted to some but not all TLRs (Lachmandas et al., 2016). This suggests that bacterial infection might induce context-specific metabolic adaptations in macrophages, different from what has been reported with the LPS challenge model.

In parallel to enhanced aerobic glycolysis, Tannahill et al. were first to show that LPS-activated murine BMDM accumulate succinate (Tannahill et al., 2013). Succinate enhances the activity of SDH (CII), which overloads coQ with electrons, forcing the electrons to flow backwards to CI in a process referred to as reverse electron transport (RET). RET was proposed to trigger CI-dependent generation of mitochondrial ROS (mROS), which has been linked to the induction of pro-inflammatory gene expression (Mills et al., 2016) (**Figure 2A**). In line with these findings, Garaude et al. showed that murine BMDM challenged with live bacteria remodel their ETC, decreasing CI assembly and switching to CII preferential utilization. This switch was dependent on sensing bacterial viability by TLRs and the NLRP3 inflammasome and was mediated by phagosomal NADPH oxidase and the ROS-dependent tyrosine kinase FGR phosphorylating and activating SDH (CII) (Garaude et al., 2016). Reciprocally, inflammasome activation was driven by CII, as its inhibition diminished the production of IL-1β (while increasing IL-10) (**Figure 2A**) (Garaude et al., 2016). Importantly, inhibition of CII rendered mice more susceptible to infection with *S. typhimurium* or *E. coli* (Garaude et al., 2016). More recently, the role of RET and CI as the source of mROS has been challenged (Cameron et al., 2019). CIII was shown to produce mROS in LPS-stimulated murine BMDM. Inhibition of CIII which is hypothesized to drive RET, reduced rather than increased inflammatory cytokine production. Consistently, deletion of the CI subunit NDUFS4 leads to systemic inflammation in *Ndufs4^-/-^* mice (Jin et al., 2014).

One of the effects of mROS accumulation in LPS-activated murine BMDM is the depletion of the cellular NAD^+^ pool, leading to inhibition of NAD^+^ dependent mitochondrial respiration. On one hand, NAD^+^ is consumed by poly(ADP-ribose) polymerase activation in response to mROS-induced DNA damage (Cameron et al., 2019). On the other hand, the *de novo* synthesis of NAD^+^, derived from tryptophan metabolism *via* the kynurenine pathway, is inhibited in response to TLR4 engagement as revealed by Isotype tracer studies (Minhas et al., 2019). To replenish NAD^+^ levels and sustain GAPDH activity in glycolysis, TLR4 quickly induces the NAD^+^ salvage pathway by upregulating the expression of nicotinamide phosphoribosyltransferase (NAMPT) (Cameron et al., 2019). Together, these studies demonstrate that PRR-stimulated murine BMDM convert their mitochondria from ATP- to ROS-producing factories, which depletes NAD^+^ levels and inhibits mitochondrial respiration while promoting glycolysis. Of note, NAD^+^ also controls the Sirtuins, a family of NAD^+^ dependent type III deacetylases that modulate inflammation by deacetylating various substrates, including transcription factors involved in macrophage activation such as NF-κB and AP-1 (Galli et al., 2011). A competition among the SIRT family members can fine-tune the macrophage inflammatory response, as recently shown for SIRT5 using murine peritoneal macrophages (Qin et al., 2017).

### Itaconate, an Anti-Inflammatory and Bactericidal Derivative of the Tricarboxylic Acid Cycle

As a consequence of the break in the TCA cycle at the level of ACO2, inflammatory macrophages convert citrate to itaconate through the mitochondrial enzyme immune-responsive gene 1 (IRG1)-mediated decarboxylation of cis-aconitate, as shown using murine peritoneal macrophages (Michelucci et al., 2013). Itaconate blocks SDH, resulting in impaired succinate oxidation, diminished oxygen consumption (Németh et al., 2016) and reduction in the levels of inflammatory cytokines (IL-1β, IL-18, IL-6, IL-12), NO and HIF-1α. Mechanistically, itaconate inhibits inflammation *via* the alkylation and inactivation of KEAP1 (Kelch-like ECH-associated protein 1) (Mills et al., 2018), which is a repressor of the transcription factor Nrf2 (Bellezza et al., 2018), allowing Nrf2 to exert its anti-oxidant and anti-inflammatory effects. The anti-inflammatory property of itaconate was also observed *in vivo* for e.g. in murine models of LPS-induced lethality (Liao et al., 2019), ischemia-reperfusion injury (Lampropoulou et al., 2016), and *Mtb* infection (Nair et al., 2018). In the latter, *Irg1^-/-^* mice were shown to be more susceptible to *Mtb* infection than wild-type animals, due to a more severe immunopathology. Besides its anti-inflammatory activity, itaconate is a potent anti-bacterial, as it inhibits the key enzyme of the bacterial glyoxylate cycle isocitrate lyase, and has been shown to restrict the growth and virulence of *Mtb* (Michelucci et al., 2013), *S. typhimurium* (Michelucci et al., 2013) and *Legionella pneumophila* (Naujoks et al., 2016).

### HIF-1α and Aerobic Glycolysis Govern the Macrophage Inflammatory and Anti-Bacterial Response

HIF-1α is a master transcription factor best known for its role in cellular adaptation to hypoxia. In bacterial infection, HIF-1α levels are upregulated at the transcriptional level by NF-κB, as shown in murine BMDM infected with group A *Streptococcus* or *Pseudomonas aeruginosa* or in LPS challenge of the murine macrophage cell-line (RAW264.7) (Rius et al., 2008). The stability of HIF-1α is also regulated at the post-translational level. The latter is induced by succinate accumulation in inflammatory macrophages. On one hand, succinate inhibits prolyl hydroxylases (PHD) (Tannahill et al., 2013), a family of α-KG-dependent dioxygenases (α-KGDD) involved in HIF-1α degradation at steady state (Bruick and McKnight, 2001). PHDs are also indirectly inhibited by mROS leading to HIF-1α stabilization (Mills et al., 2016). On the other hand, succinylation of the glycolysis rate limiting enzyme pyruvate kinase M2 (PKM2) converts it from an active homotetramer into an inactive monomer/dimer that binds to and activates HIF-1α (Palsson-McDermott et al., 2015). Among the key inflammatory genes with a HIF-1α response element is the gene encoding pro-IL-1β. Thus HIF-1α couples the metabolic shift to aerobic glycolysis in murine BMDM to the induction of IL-1β-mediated inflammatory response (**Figure 2A**). In chronic infection with some intracellular bacteria, HIF-1α stabilization leads to reduced citrate levels, and such a nutritional depletion prevents bacterial replication but without impacting bacterial survival leading to bacterial persistence (Hayek et al., 2019). This was demonstrated for *Coxiella burnetii*, the causative agent of Q fever, using human monocyte-derived macrophages (hMDM) and in murine BMDM and for *L. Pneumophila*, the causative agent of Legionnaires’ pneumonia, in a murine BMDM infection model.

### The Role of Glutaminolysis, α-KG, and the Hexosamine Biosynthetic Pathway (HBP) in M2 Macrophage Polarization

Using metabolomics and transcriptomics approaches on murine BMDM cultured under LPS+IFNγ elicited M1 or IL-4-induced M2 polarizing conditions, Jha et al. identified different metabolic pathways important for each macrophage state. In particular, an enrichment of effectors and metabolites of the glutaminolysis and the HBP was observed in M2 macrophages. Concordantly, inhibition of N-glycosylation or glutamine deprivation reduced M2 polarization (Jha et al., 2015). The same was seen with inhibition of the glutaminase Gls1, which was demonstrated to promote M2 polarization through elevation of α-KG production and epigenetic upregulation of M2-associated genes by Jumonji domain-containing protein D3 (JMJD3) (Liu et al., 2017), a histone demethylase belonging to the α-KGDD superfamily (Loenarz and Schofield, 2008). Glucosamine treatment, which engages the HBP, suppressed LPS-induced proinflammatory gene expression in BMDM and improved clinical outcomes in the cecal ligation and puncture (CLP) mouse model of sepsis (Hwang et al., 2019) (**Figure 2B**). Conversely, myeloid-specific knockout of murine *Ogt*, which encodes the HBP effector enzyme O-GlcNAc transferase (OGT) led to heightened susceptibility to LPS-induced septic shock, mediated by exacerbated macrophage inflammation (Li et al., 2019a; Li et al., 2019b).

### Metabolic Epigenetic Control of Inflammation and Trained Immunity

As mentioned earlier, α-KG exerts important epigenetic regulation of murine BMDM polarized to the M2 phenotype through the histone demethylase activity of JMJD3 (Liu et al., 2017). Another layer of control of macrophage inflammatory response is mediated by acetyl-CoA production and histone acetylation. Using metabolic tracing of glucose and glutamine and metabolic assays, Lauterbach et al. recently demonstrated that TLR4 stimulation of murine BMDM activates ATP-citrate lyase, which converts citrate pumped out of the mitochondria to acetyl-CoA in the cytosol. Acetyl-coA is then used for histone acetylation and activation of several inflammatory gene loci, including the *Il-12* locus, linking cellular metabolism to epigenetic activation of innate immunity (Lauterbach et al., 2019) (**Figure 2B**). A new post-translational modification (PTM) of histone lysine residues, derived from lactate and referred to as lactylation, has recently been added to the metabo-epigenetic armamentarium of macrophage regulation (Zhang et al., 2019). Interestingly, this PTM appears to occur later in the course of murine BMDM polarization than histone acetylation, upregulating genes involved in wound healing (e.g. Arg1) presumably to restore homeostasis (Zhang et al., 2019).

The immune tolerance state observed in macrophages following prolonged exposure to LPS, which provides a model to study immune paralysis, as observed in sepsis (Kumar, 2018), is similarly controlled by chromatin remodeling. Seeley et al. showed that sustained LPS promoted murine BMDM tolerance by inhibiting STAT1/2-dependent upregulation of inflammatory genes. Prolonged LPS treatment induced two microRNAs, miR-221 and miR-222, that inhibited the chromatin remodeling complex SWI/SNF by targeting its core component brahma-related gene 1 (Brg1). Interestingly, expression of miR-221/-222 correlated with increased organ damage in sepsis patients, and may potentially serve as a biomarker of sepsis-related immune paralysis (Seeley et al., 2018). Beyond the regulation of innate immunity, the metabolic-epigenetic crosstalk exerts a key role in the establishment of trained immunity. The two main epigenetic marks linked to trained immunity are histone methylation (H3K4me3) in promoters and histone acetylation (H3K27ac) in distal enhancers of poised innate immunity genes within specific loci in the genome. In human monocytes stimulated with β-glucan (to induce trained immunity), Arts et al. showed that cholesterol synthesis, aerobic glycolysis and glutamine anaplerotic use in the TCA cycle, lead to fumarate accumulation, which induces epigenetic rewiring of macrophages by inhibiting the histone demethylase lysine demethylase 5 (KDM5) (**Figure 1B**). Inhibition of glutaminolysis and cholesterol synthesis in mice reduced trained immunity induction *in vivo* (Arts et al., 2016). β-glucan inhibits IRG1 thus limiting itaconate inhibition of SDH; as a consequence succinate is converted to fumarate in β-glucan-trained human MDM (Dominguez-Andres et al., 2019).

## Bacteria Rewire Macrophage Metabolism as a Strategy to Grow and Evade Innate Immunity

**Figure 3:**
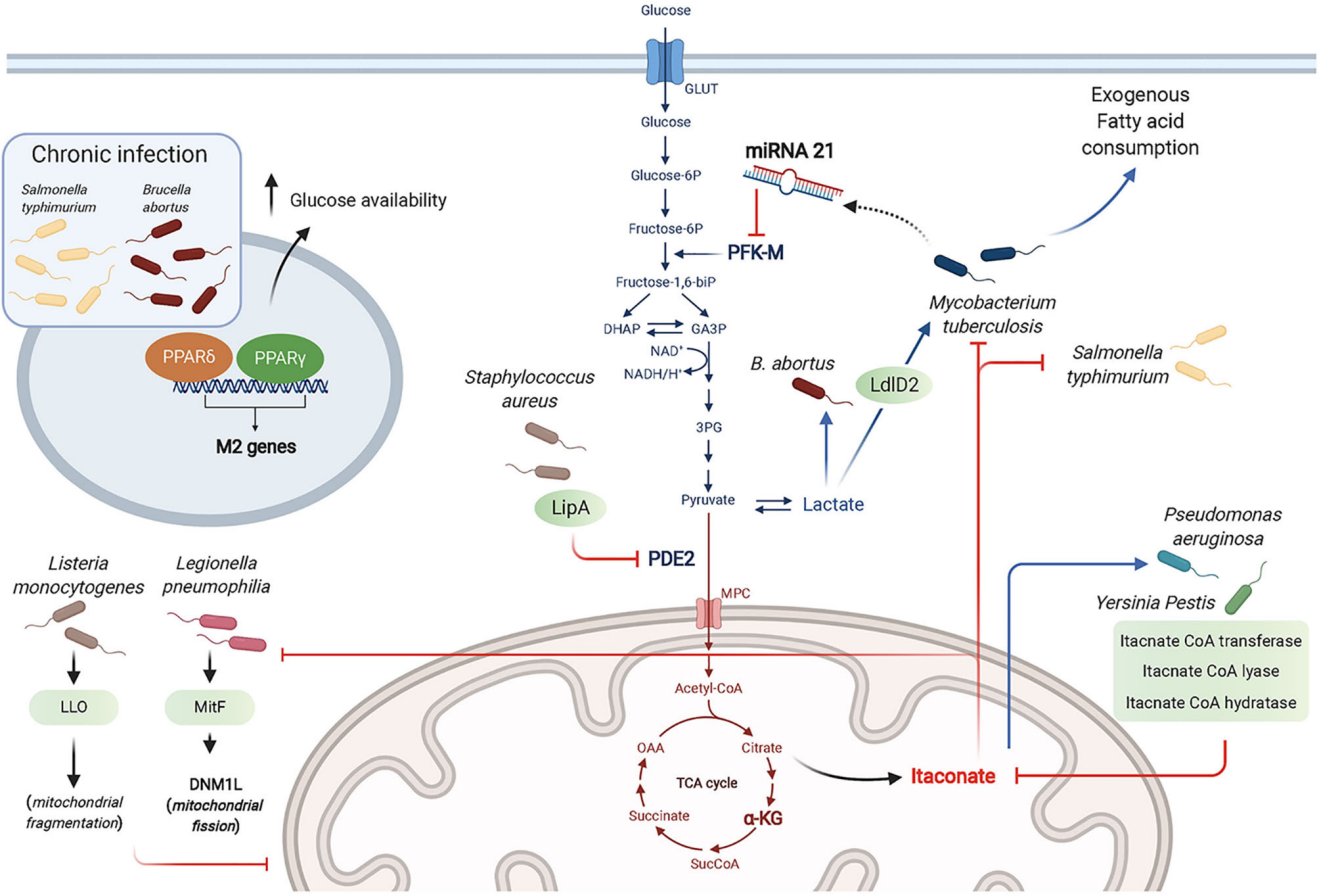
Bacterial strategies targeting macrophage metabolism toward a successful infection. Bacteria manipulate metabolic pathways to enhance the necessary nutrient resources required for their survival, including glucose and lactate as carbon sources. The antibacterial effects of itaconate is countered by some bacteria through expression of itaconate degradative enzymes. Bacteria can “hide” from innate sensors by altering host metabolic enzymes (e.g. PDE2 conversion into lipoyl-E2-PDH that blocks TLR1/2 stimulation by bacterial lipopeptides). Chronic infection by intracellular bacteria is promoted through the actions of PPARδ and PPARγ that promote a wound healing phenotype and enhance glucose availability for the bacteria.

Some intracellular bacterial pathogens evolved multiple and overlapping mechanisms to survive within the threatening environment of a macrophage (**Figure 3**). These include exploiting macrophage metabolic resources to survive and rewiring macrophage metabolism to attenuate bacterial sensing and innate anti-bacterial defenses. Furthermore, some bacteria adapt to macrophage innate immunity responses to tolerate antibiotics.

### Exploiting the Macrophage Glycolytic Pathway as a Nutrient Source

Among the intracellular bacteria shown to rewire macrophage metabolism toward aerobic glycolysis are *L. pneumophila*, *Brucella abortus*, *Mtb* and *Listeria monocytogenes*. While *L. pneumophila* depends on serine metabolism in its exponential growth phase, it switches to glycerol and glucose use in the post-exponential phase (Hauslein et al., 2017; Oliva et al., 2018). *L. pneumophila* establishes a permissive niche by impairing macrophage OXPHOS in a type IV secretion system (T4SS)-dependent manner (Escoll et al., 2017). Using human MDM and the murine RAW264.7 macrophage cell-line, Escoll et al. showed that *L. pneumophila* triggered mitochondrial fission through the secreted effector MitF, a Ran GTPase activator that interacts with host DNM1L, a GTPase involved in mitochondrial fission. In parallel, *L. pneumophila* enhances glycolysis in a T4SS-independent manner, although the mechanism controlling this pathway has not been determined. Using macrophages derived from the human monocytic cell-line THP-1, Czyż et al. showed that *B. abortus* increased macrophage aerobic glycolysis to promote its survival, which depended on lactate as its sole carbon source. This was demonstrated using a *Brucella* strain deficient in lactate dehydrogenase or by pharmacological inhibition of host glycolysis, both resulting in impaired *Brucella* growth (Czyz et al., 2017). *Mtb* was reported to use lactate instead of pyruvate as a carbon source. Using WT and KO mutant strains, Billig et al. showed that this ability to use lactate relied on oxydation by the L-lactate dehydrogenase LldD2. ^13^C tracing experiments proved that lactate was used in the bacterial TCA cycle and for gluconeogenesis *via* phosphoenolpyruvate carboxykinase. This pathway was key for *Mtb* intracellular survival in human macrophages (Billig et al., 2017). The cytosolic bacteria *Listeria monocytogenes* poorly metabolizes lactate and pyruvate, but relies on glycerol and glucose-6-phosphate for energetic and anabolic needs, respectively (Grubmuller et al., 2014). Through its toxin Listeriolysin O (LLO), *L. monocytogenes* induces transient mitochondrial fragmentation (Stavru et al., 2011), and takes advantage of the increased glycolysis elicited in inflammatory macrophages to proliferate (Gillmaier et al., 2012).

### Dampening Macrophage Glycolysis to Promote Chronic Infection

*B. abortus* chronic intracellular infection preferentially occurs in alternatively activated or M2 macrophages, relying on PPARγ that contributes to increased glucose availability for the bacteria (Xavier et al., 2013). Similarly, *S. typhimurium* hijacks glucose from the host cell requiring the transcription factor PPARδ to sustain chronic infection as shown using murine BMDM (Eisele et al., 2013). Unlike the findings by Billig et al. described above (Billig et al., 2017), Cumming et al. demonstrated that enhanced aerobic glycolysis was only observed with *M. bovis* BCG or dead *Mtb*. In contrast, live virulent *Mtb* directed human macrophage metabolism to exogenous fatty acid consumption instead of glucose while globally reducing both glycolysis and the TCA cycle. *Mtb* thus reprograms macrophage metabolism into a “quiescent state” to facilitate its intracellular survival (Cumming et al., 2018). Among the mechanisms by which *Mtb* dampens macrophage glycolysis is through the induction of microRNA-21 (miR-21) that targets the glycolysis limiting enzyme phosphofructokinase-M (PFK-M). Using WT or miR-21 deficient mice and *in vitro* assays with human and mouse macrophages, Hackett et al. demonstrated that this anti-inflammatory miR-21 dampened glycolysis and ultimately decreased IL-1β production, promoting bacterial growth. Interestingly, IFNγ secreted in response to *Mtb* infection counters miR-21 induction, restoring the macrophage anti-bacterial response (Hackett et al., 2020).

### Countering the Anti-Bacterial Effect of Itaconate

Several bacteria, including *Yersinia Pestis* and *P. aeruginosa* degrade itaconate as a common survival strategy, by expressing three enzymes, namely itaconate coenzyme A (CoA) transferase, itaconyl-CoA hydratase, and (S)-citramalyl-CoA lyase (Sasikaran et al., 2014). Furthermore, *P. aeruginosa* exploits itaconate as a carbon source allowing it to produce biofilm as in cystic fibrosis patients lungs. Riquelme et al. recently reported that itaconate-adapted *P. aeruginosa* accumulate mutations in the LPS-assembly protein IptD, and upregulate extracellular polysaccharides, which in turn promotes itaconate production by macrophages in a feedforward mechanism (Riquelme et al., 2020).

### Targeting Metabolic Effectors to “Hide” From Macrophage Pattern Recognition Receptors and to Tolerate Antibiotics

Using murine BMDM, Grayczyk et al. determined that *S. aureus* is able to secrete a lipoic acid synthetase, LipA, that modifies pyruvate dehydrogenase E2 subunit (PDE2) by adding a lipid moiety, lipoic acid. This yields the secreted metabolic protein lipoyl-E2-PDH that blocks TLR1/2 stimulation by bacterial lipopeptides. Altogether, these data suggest a key role for LipA in bacterial escape from innate immunity (Grayczyk et al., 2017). *S. aureus* also benefits from host-derived ROS to tolerate antibiotics. Rowe et al. showed that ROS attenuated the metabolism of *S. aureus* by attacking iron-sulfur (Fe-S) clusters-containing proteins including bacterial TCA cycle enzymes, namely SDH and acotinase. This metabolic state confers *S. aureus* resistance to killing by multiple antibiotics, highlighting a situation where innate immunity is exploited by the bacteria for a successful infection (Rowe et al., 2020).

## Conclusion and Future Perspectives

The last decade has witnessed an impressive growth in the understanding of the intricate immunometabolic network governing macrophage activation in bacterial infections. Furthermore, several studies have now described strategies used by intracellular bacterial pathogens to survive in macrophages, evade innate immunity and establish a chronic infection. These advances provide exciting perspectives for developing new therapies targeting macrophage metabolic effectors to treat infectious and inflammatory diseases. Notable example of currently approved anti-inflammatory drugs that target metabolic effectors include methotrexate, rapamycin and metformin, that respectively inhibit dihydrofolate reductase, mammalian target of rapamycin (mTOR), and CI. Additional metabolic modulators include dimethyl fumarate (DMF) that inhibits KEAP1, NF-κB and the inflammasome, among others, and TEPP-46 that promotes PKM2 tetramerization [reviewed in (Palsson-McDermott and O’Neill, 2020)]. However, to expand this armamentarium, the focus must be steered away from tissue culture models of macrophage infection and *in vitro* macrophage polarization studies to fully grasp organ-specific immunometabolic mechanisms of macrophages of different lineages involved in fighting or containing bacterial infections. Exploring the efficacy of new immunometabolic modulatory drugs might provide urgently needed therapeutic options for emerging infectious diseases or those resistant to approved therapies.

## Author Contributions

GG and MS both wrote the text and illustrated the figures. All authors contributed to the article and approved the submitted version.

## Funding

This study was funded by IDEX Bordeaux. MS is funded by the ARC Foundation, IDEX Bordeaux, The New Aquitaine Region and SIRIC BRIO. GG is funded by a studentship from the University of Bordeaux Hospital.

## Conflict of Interest

The authors declare that the research was conducted in the absence of any commercial or financial relationships that could be construed as a potential conflict of interest.
